# How useful is the tuberculin skin test for the detection of active tuberculosis: Assessing diagnostic accuracy metrics and selection of best cut-off value through a large Tunisian multicenter case-control study

**DOI:** 10.12688/f1000research.138211.1

**Published:** 2023-10-10

**Authors:** Mariem Nouira, Hazem Ben Rayana, Samir Ennigrou

**Affiliations:** 1 Faculty of Medicine of Tunis, Tunis, Tunisia

**Keywords:** tuberculosis; tuberculin skin test, diagnostic accuracy, sensitivity, specificity, Predictive Value of Tests, Receiver operating characteristic curve, case-control study

## Abstract

Background and aim:

During the past decade, the frequency of extrapulmonary forms of tuberculosis (TB) has increased. These forms are often miss-diagnosed. This statement of the TB epidemiological profile modification, conduct us to reflect about the utility of the Tuberculin Skin Test (TST) in active TB detection. This study aimed to evaluate the diagnostic accuracy performance of the TST for active tuberculosis detection.

Methods:

This was a case-control, multicenter study conducted in 11 anti-TB centers in Tunisia (June-November2014). The cases were adults aged between 18 and 55 years with newly diagnosed and confirmed tuberculosis. Controls were free from tuberculosis. A data collection sheet was filled out and a TST was performed for each participant.

Diagnostic accuracy measures of TST were estimated using Receiver Operating Curve (ROC) curve and Area Under Curve (AUC) to estimate sensitivity and specificity of a determined cut-off point.

Results:

Overall, 1050 patients were enrolled, composed of 336 cases and 714 controls. The mean age was 38.3±11.8 years for cases and 33.6±11 years for controls.

The mean diameter of the TST induration was significantly higher among cases than controls (13.7mm vs.6.2mm; p=10 ^-6^). AUC was 0.789 [95% CI: 0.758-0.819; p=0.01], corresponding to a moderate discriminating performance for this test. The most discriminative cut-off value of the TST, which was associated with the best sensitivity (73.7%) and specificity (76.6%) couple was   ≥ 11 mm with a Youden index of 0.503. Positive and Negative predictive values were 3.11% and 99.52%, respectively.

Conclusions:

TST could be a useful tool used for active tuberculosis detection, with a moderate global performance and accepted sensitivity and specificity at the cut-off point of 11 mm. However, it cannot be considered as a gold standard test due to its multiple disadvantages.

## Introduction

The tuberculosis (TB) epidemic poses a serious public health problem, with high associated morbidity and mortality rates worldwide. ^1^ It has been estimated that almost a quarter of the total world’s population is infected with latent TB infection and approximately 10 million have developed active TB, with about 1.6 million attributed deaths in 2021, all over the world. ^2^

According to the World Health Organization (WHO) estimations, diagnosing and treating TB saved 66 million lives between 2000 and 2020. ^2^ Therefore, there is a need to improve the TB diagnostic procedure. It is a priority requirement to dispose of an accurate diagnostic tool for early detection and appropriate treatment, and set up timely control measures to limit the spread of the infection. ^3^ However, there is no international agreement about a gold-standard test for detection of active nor latent TB. ^3^^,^^4^

The detection of active TB using a simple, rapid, and inexpensive test, like the tuberculin skin test (TST), in diagnosing TB, may help to guide easier clinical diagnosis of disease.

The TST or Mantoux test is an old, widely used test for TB infection screening and for estimating the delayed hypersensitivity reaction induced by BCG vaccination. ^5^^,^^6^

Over the past decade, the frequency of extrapulmonary forms of TB has increased. ^7^^,^^8^ These forms, especially lymph node TB type, are often miss-diagnosed, due to unspecific symptoms, and pose a diagnostic challenge for clinicians, requiring the use of invasive methods for diagnostic confirmation. ^9^^,^^10^

Tunisia is a country of intermediate TB endemicity with an estimated incidence of 29/100,000 inhabitants in 2017. Pulmonary TB constituted 38% of all forms of the disease in 2017, while extra-pulmonary TB represents 62% of cases. The frequency of lymph node forms is relatively high, with a constant increase from 2.3/100,000 in 1993 to 18/100,000 in 2017. ^11^

These TB epidemiological profile modifications in Tunisia (the increase in the frequency of lymph node forms at the expense of pulmonary forms) and all arguments cited above, leads us to reflect on the utility and the performance of the TST for active TB detection.

The utility of the TST for the detection of latent TB infection was largely discussed and debated in the literature, ^12^^,^^13^ but its utility in diagnosing active TB has not been discussed enough in previous studies.

Therefore, we aimed, through this multicenter case-control study, to evaluate the diagnostic accuracy of the TST for the diagnosis of active TB, and to select its best cut-off value, using the ROC curve and Fagan’s Nomogram methodology.

## Methods

### Study design

This was a case-control, multicenter study for a purpose of a diagnostic test evaluation, conducted in 11 anti-TB centers in Tunisia, during the period from June to November 2014.

### Study population

*Inclusion and exclusion criteria*

Included TB cases were adult patients aged between 18 and 55 years who were newly diagnosed with TB. They had confirmed TB diagnosis (pulmonary and extra-pulmonary forms of tuberculosis) based on a range of anamnestic, clinical, chest X-ray, bacteriological arguments. They were recruited from 11 anti-TB centers from the north, center and south of Tunisia (Ariana, Tunis, Sfax, Gafsa, Ben Arous, Bizerte, Sousse, Kairouan, Sidi Bouzid, Kasserine, Tataouine), at the first-time delivery of their anti-TB treatment.

Controls were recruited from basic health centers and from district hospitals, located in the same geographical zone of the anti-TB centers (in which cases were recruited) and during the same study period. The gender distribution frequency was approximately the same as that of TB cases. Overall, the same proportion of men and women in TB cases and in controls was respected. All the included controls had no clinically manifested active TB; they did not present any respiratory or extra-respiratory symptoms that could be of TB origin.

*Exclusion criteria*

Were excluded from the study:

‐ TB cases already treated for pulmonary or extra-pulmonary TB.
‐ TB cases and controls with a pathological condition that may lead to tuberculin anergy: acute viral infections (measles, infectious mononucleosis, influenza), lymphomas, neoplastic pathologies, sarcoidosis, severe bacterial infection, HIV infection, among others.
‐ TB cases and controls having undergone immunosuppressive treatment, corticosteroid therapy for more than one month or vaccination with live attenuated vaccines two months prior to the test.
‐ TB cases and controls with a history of known allergic reaction to one of the components of TST or during a previous administration.

### Data collection

Data was collected using a standardized questionnaire for all participants including information regarding demographic variables (age, sex, educational level), as well as medical history of any pathological condition that may lead to tuberculin anergy, the status of participant (case or control) and the type of TB infection for cases (pulmonary or extra-pulmonary), as well as the date of administration and lecture of the TST result.

*Tuberculin skin testing (TST)*

It should be noted that the TST was performed for all participants, cases, and controls, not as an investigator-mandated intervention, but as part of the normal process of diagnosing the etiology of their disease.

The test was performed using the Mantoux method, in all participants (cases and controls) by injecting 0.1 mL of tuberculin solution (tuberculin PPD [Purified Protein Derivative RT23 from Copenhagen]), strictly intradermally, on the forearm away from any other scar. ^6^

The TST results were read 72 hours after administration by the same trained investigator. Diameter of induration was determined by calculating the average of the transverse and longitudinal diameter of induration (in mm). ^6^

### Statistical analysis

Continuous and categorical variables were summarized using the mean (± standard deviation) and relative frequencies (expressed into percentage), respectively. Pearson’s Chi-square test and Student’s T test were used for comparing two percentages or two means for independent samples, respectively. For all statistical tests, the significance level adopted was 0.05. All statistical analyses were performed using SPSS version 23.0 software.

*Sensitivity and specificity*

To assess the performance of the TST, we first calculated the sensitivity and specificity of the TST induration diameter as well as the Youden index for different possible thresholds (from a diameter of TST ≥ 5 mm to a TST diameter ≥ 15 mm).

The calculation of the 95% confidence intervals (95% CI) of the sensitivity and specificity was done using an Excel calculator and using the properties of the exact binomial distribution.

Sensitivity (Se) was defined as the proportion (ranging from 0 to 1) of tuberculosis cases who tested positive (true positives = TP) with TST. The proportion of cases which were not identified using TST were false negative (FN) results. ^14^

Specificity (Sp) was defined as the proportion (ranging from 0 to 1) of controls who tested negative for the disease (true negatives = TN) with TST. The proportion of controls which were tested positive using TST were false positive (FP) results. ^14^^,^^15^

The two intrinsic qualities of the test, sensitivity, and specificity, were aggregated into an index, known as the Youden Index noted J such that: J = (Se + Sp) – 1.

Youden index varies between -1 and +1; a value less than or equal to 0 reflects the diagnostic ineffectiveness of the test. The test performance is better when its Youden index is close to 1. ^16^

Positive and negative predictive values (PPV and NPV) (or post-test probabilities) of TST were also determined for different possible TST thresholds by two different methods, using the properties of Bayes’ theorem ^17^ and using Fagan’s nomogram graph. ^18^

The predictive values were deducted from Fagan’s nomogram graph based on the TB prevalence (or pre-test probability) according to TB patients’ data from three university hospitals in Tunis, Tunisia (The Rabta Hospital, Charles Nicolle Hospital and Abderrahmane Mami hospital). The estimated TB prevalence was around 1% in 2016.

The positive and negative likelihood ratios (LR) were also determined for different possible thresholds of the TST, and their 95% confidence intervals (95% CI) were calculated using the Excel calculator.

The positive LR (varies from 1 to +∞) indicates that the TST a test is more discriminating tuberculosis cases from non-cases when it is far from 1 and the specificity approaches 1.

The negative LR (varies from 0 to 1) indicates that the TST is better able to discriminate TB cases from non-cases when it is closer to 0 and the sensitivity approaches to 1.

*ROC and area under the curve (AUC)*

The overall discriminative performance of the TST was evaluated using the ROC curve, ^19^ to determine the optimal cut-off value of the best couple (sensitivity, specificity).

We established a ROC curve, first for all participants (age, sex and site of infection combined), then according to site infection (pulmonary and lymph node tuberculosis) and according to age groups.

The AUC was calculated for each ROC curve and presented with their 95% confidence intervals (95% CI). The AUC can vary between 0.5 and 1. The closer it is to 1, the better the discriminating ability or overall diagnostic value of the TST.

### Ethical considerations

All included participants were informed about the purpose of the study and have give their written informed consent to participate to the study and carrying out the TST. They were also informed about their right to refuse participation or drop out at any moment of the study collection. All collected information and data analysis was confidential and anonymous during and after data collection.

In Tunisia, there was only one national ethics committee at the time of study (2014) which was in charge of requests for clinical trial type studies only. Since no blood samples were taken or procedures performed on the participants for research purposes, and since the normal process of diagnosis and management of all patients was respected without any intervention (the TST was practiced for all participants for a diagnostic purpose and not for a research purpose), we did not submit our study to that committee at the time. The study was retrospectively approved, on August 2023 by the ethics committee of the Faculty of Medicine Of Tunis under the approval number CE-FMT/2023/03/HCN/V1.

## Results

### Sociodemographic characteristics of the study population

Overall, 1050 participant were included (336 cases and 714 controls) with a mean age of 35 years. Of the cases, more than half were female (n = 179, 53.3%), sex ratio (M/F) = 0.87, with a mean (± standard deviation) age of 38.3 (±11.8) years. Of the controls, half were female (n = 358, 50.1%), sex-ratio (M/F) = 0.99, with a mean (± standard deviation) age of 33.6 (±11.0) years (see Table 1). The Bacillus Calmette-Guérin vaccination scar was present among all included controls and among most TB cases (83.8%).

**Table 1.** Sociodemographic and clinical characteristics of the cases (n = 336) and controls (n = 714) included.

Characteristics		Cases n (%) mean( ±SD)	Controls n (%) mean (±SD)
**Sex**	Male	157 (46.7)	356 (49.9)
	Female	179 (53.3)	358 (50.1)
**Age** (years)		38.3 (11.8)	33.6 (11.0)
**Education level**	Analphabetic	56 (16.7)	29 (4.1)
	Primary/Secondary	233 (69.3)	451 (63.1)
	University	47 (14.0)	234 (32.8)
**Site of infection** (for cases)	Pulmonary	121 (36.0)	
	Extra-Pulmonary		
	Lymph node	180 (53.6)	
	Other	35 (10.4)	

### TST induration diameter results

**Figure 1.**
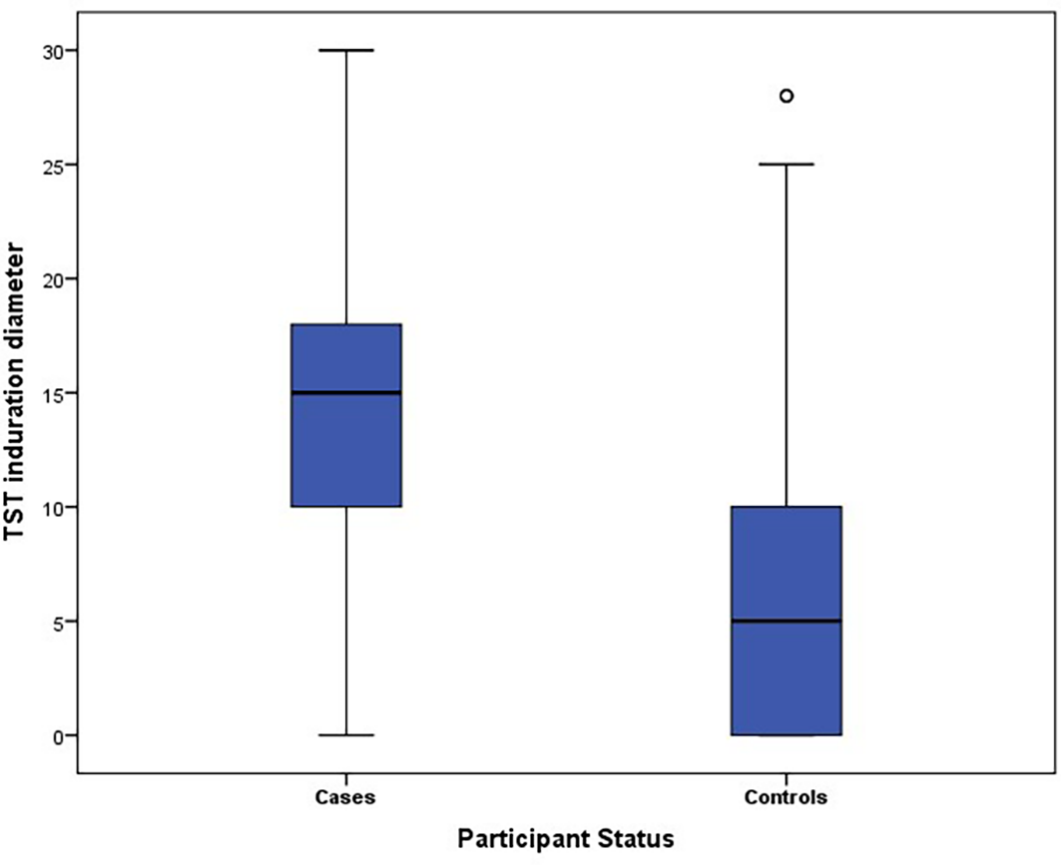
Box plot of TST diameter for cases and controls.

The mean diameter of the TST induration was significantly higher among cases than controls (13.7 mm (SD: 0.7 mm) *versus* 6.2 mm (SD: 6.4 mm); p = 10 ^-6^) (see Figure 1).

There was no significant difference in the mean diameter of the TST induration between male and female participants (8.5 mm (SD: 6.9 mm) *versus* 8.7 mm (SD: 7.9 mm); p = 0.6).

**Figure 2.**
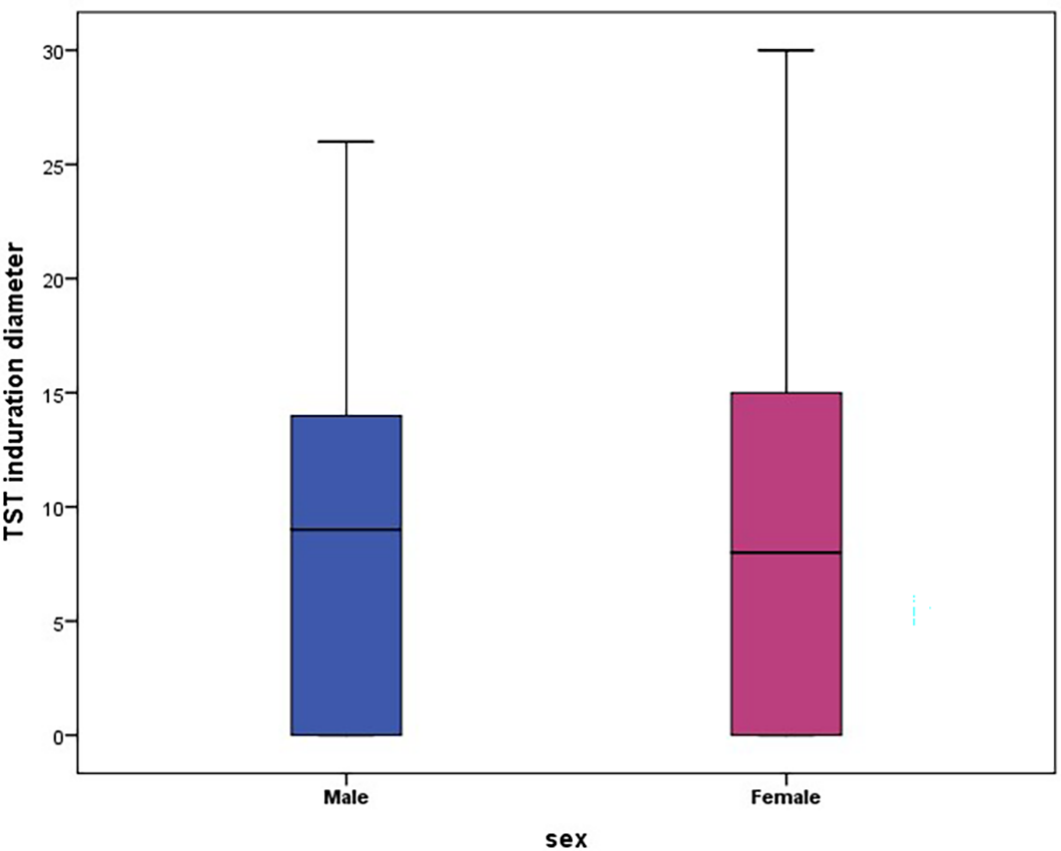
Box plot of TST diameter by sex.

The TST induration diameter disaggregated by sex and by participant status is represented in Table 2 and Figure 2.

**Table 2.** The tuberculin skin test induration diameter disaggregated by sex (male versus female) and by participant status (cases versus controls).

TST induration diameter (mm)	Male (N = 513)		Female (N = 537)	
	Cases (N = 157)	Controls (N = 356)	Cases (N = 179)	Controls (N = 358)
Mean	13.5	6.2	13.8	6.1
SD	6.1	6.1	7.6	6.7
Median	14	6	15	5.0
IQR [Q1–Q3]	[11–17]	[0–10]	[10–20]	[0–10]
Total Mean (±SD)	8.5 (±6.9)		8.7 (±7.9)	
Total Median	9.0		8.0	
Total IQR	[0–14]		[0–15]	

TST: tuberculin skin test; SD: standard deviation; IQR: inter quartile range.

### ROC curve

**Figure 3.**
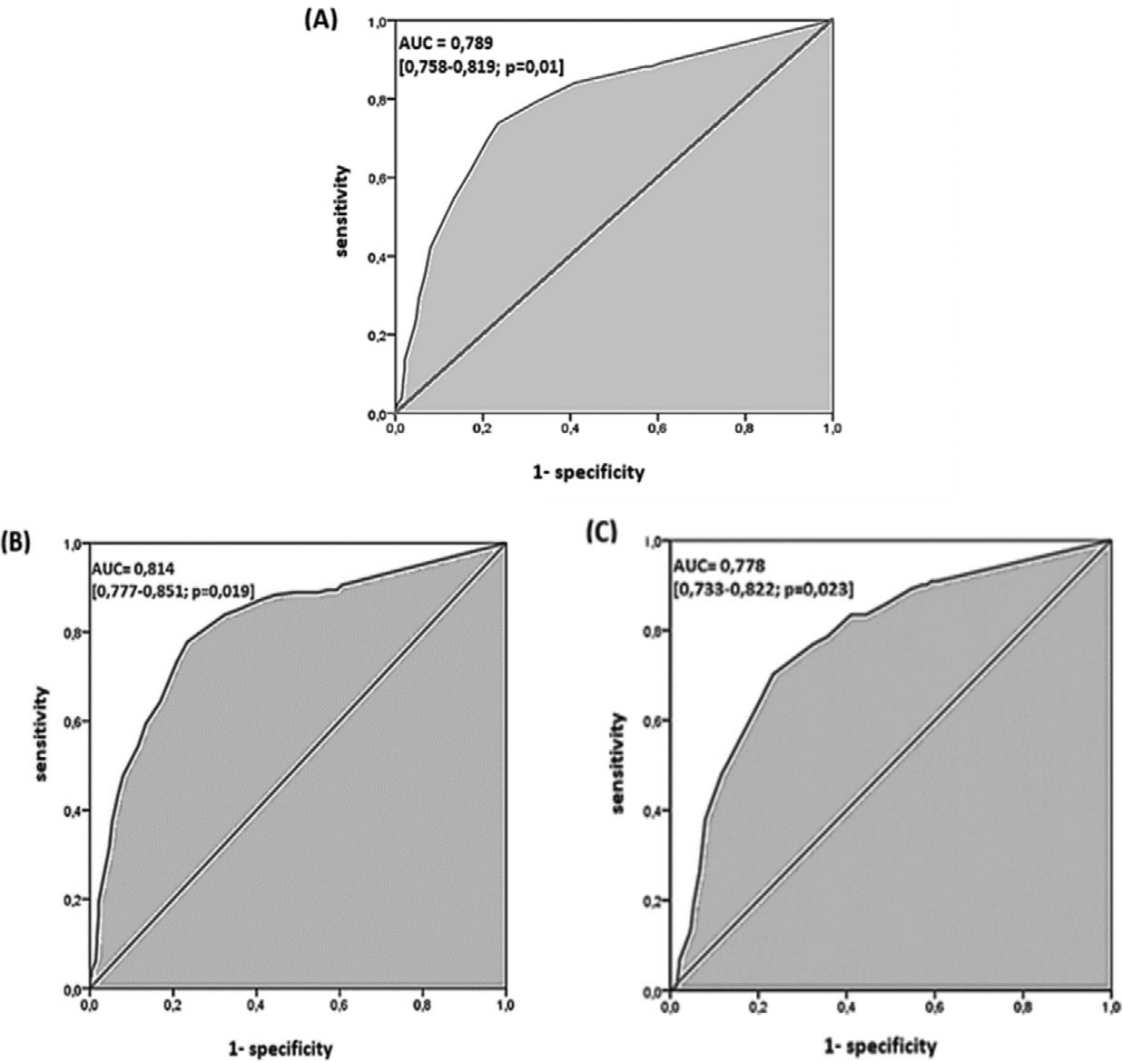
Receiver operating characteristic curve of the tuberculin of skin test in diagnosing tuberculosis. (A) All 1050 participants. (B) 180 cases of lymph node tuberculosis. (C) 121 cases of pulmonary tuberculosis.

**Figure 4.**
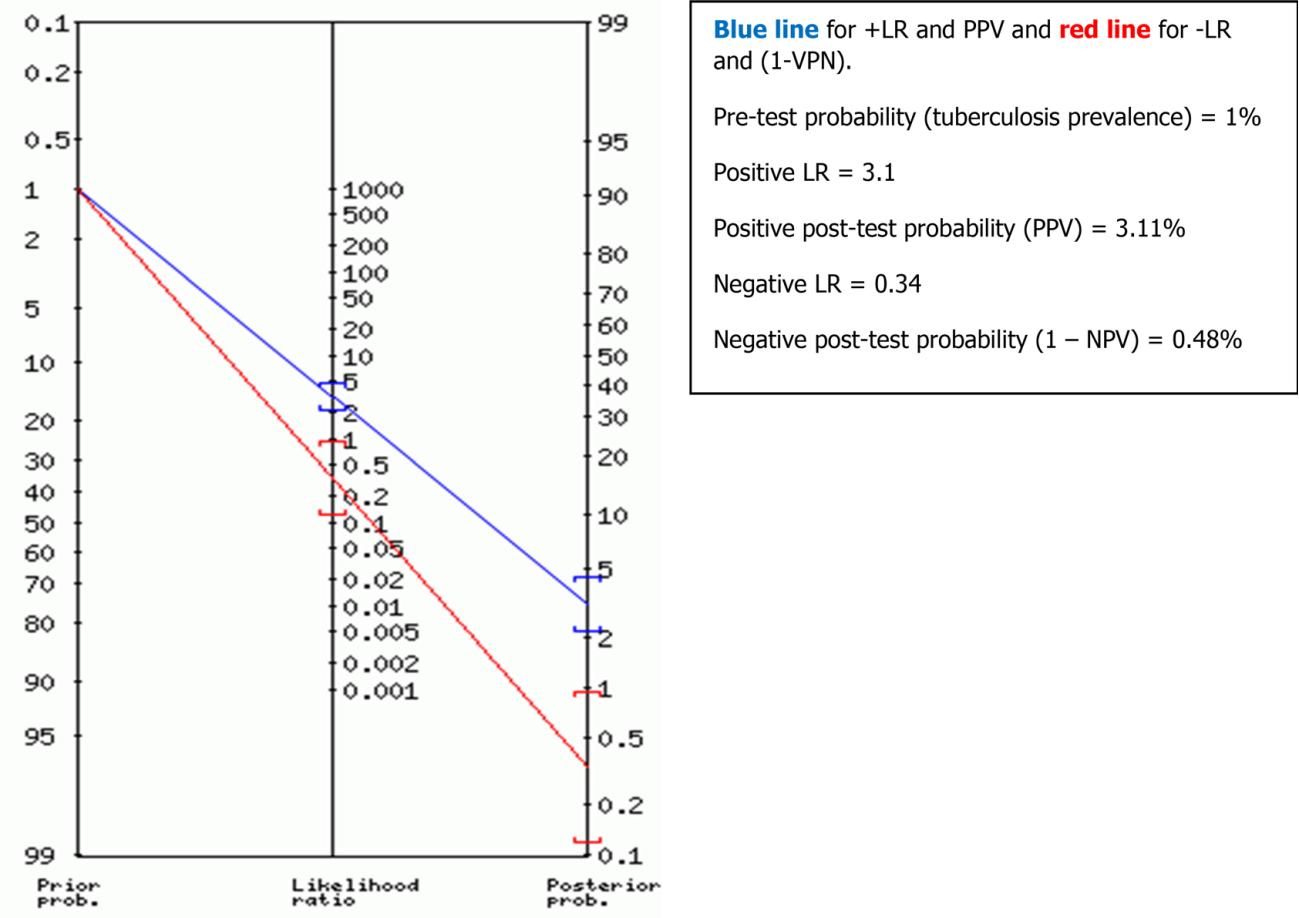
Positive predictive value (PPV) and negative predictive value (NPV) determined using Fagan's nomogram for the best selected threshold value of TST induration greater than or equal to 11 mm, for all participants (n = 1050).

For the global performance of TST among all the 1050 participants, the best discriminant cut-off value of TST was when the induration diameter of TST was ≥11 mm, with a sensitivity and specificity of 73.7% (95% CI: 68.8 % – 78.1 %) and 76.6 % (95% CI: 73.3 % – 79.5 %), respectively, with a Youden Index of 0.503 and an area under the curve (AUC) of 0.789 (95% CI: 0.758 – 0.819; p = 0.01). The sensitivity became >80% from a TST cut-off value ≥9 mm to 5 mm (see Table 3, Figure 3A). Using Fagan’s nomogram for the best selected threshold value of TST (≥11 mm), positive and negative predictive values were determined with values of 3.11% and 99.52%, respectively (see Figure 4).

**Table 3.** Sensitivity, specificity, Youden Index, positive and negative likelihood ratios values, positive predictive and negative predictive values corresponding to different possible tuberculin skin test thresholds (From 5-15 mm) for all participants (n=1050).

Diameter of TST induration ≥ (mm)	Sensitivity % [95% CI]	Specificity % [95% CI]	Youden Index	Positive LR [95% CI]	Negatif LR [95% CI]	PPV (%) [95% CI] (Bayes theorem)	NPV (%) [95% CI] (Bayes theorem)
5	87.6 [83.6 – 90.7]	45.2 [41.4 – 48.7]	0.328	1.6 [1.5 – 1.7]	0.27 [0.20 – 0.37]	1.59 [0.80 – 2.84]	99.72 [98.48 – 100.00]
6	86.1 [82.0 – 89.4]	50.8 [47.1 – 54.4]	0.369	1.7 [1.6 – 1.9]	0.27 [0.21 – 0.36]	1.71 [0.85 – 3.04]	99.75 [98.64 – 100.00]
7	85.0 [80.7 – 83.3]	55.6 [51.9 – 59.2]	0.406	1.9 [1.7 – 2.1]	0.27 [0.20 – 0.35]	1.81 [0.91 – 3.22]	99.77 [98.76 – 100.00]
8	84.1 [79.8 – 87.5]	58.9 [55.3 – 62.5]	0.430	2.0 [1.9 – 2.3]	0.27 [0.21 – 0.34]	2.07 [1.07 – 3.59]	99.36 [98.16 – 99.86]
9	81.1 [76.6 – 84.9]	64.4 [60.8 – 67.8]	0.455	2.3 [2.0 – 2.6]	0.29 [0.23 – 0.37]	2.26 [1.17 – 3.92]	99.61 [98.62 – 99.95]
10	79.4 [74.7 – 83.3]	67.6 [64.1 – 70.9]	0.470	2.4 [2.2 – 2.8]	0.30 [0.25 – 0.38]	2.40 [1.24 – 4.15]	99.63 [98.69 – 99.95]
**11**	**73.7** [68.8 – 78.1]	**76.6** [73.3 – 79.5]	**0.503**	**3.1** [2.7 – 3.6]	**0.34** [0.28 – 0.41]	**3.11** [1.67 – 5.27]	**99.52** [98.62 – 99.90]
12	69.3 [64.2 – 73.9]	79.1 [76.0 – 81.9]	0.484	3.3 [2.8 – 3.9]	0.39 [0,33-0,46]	3.12 [1.62 – 5.39]	99.55 [98.69 – 99.90]
13	61.4 [56.0 – 66.4]	83.1 [80.1 – 85.6]	0.445	3.6 [3.0 – 4.3]	0.46 [0.40 – 0.53]	3.64 [1.89 – 6.28]	99.44 [98.59 – 99.84]
14	54.9 [49.5 – 60.1]	86.6 [83.9 – 88.9]	0.415	4.1 [3.3 – 5.0]	0.52 [0.46 – 0.59]	3.90 [1.96 – 6.87]	99.35 [98.49 – 99.78]
15	50.7 [45.4 – 56.0]	88.4 [85.8 – 90.5]	0.391	4.4 [3.5 – 5.5]	0.56 [0.50 – 0.62]	4.31 [2.17 – 7.58]	99.37 [98.54 – 99.79]

TST: tuberculin skin test; CI: confidence interval; PPV: positive predictive value; PPV: negative predictive value; LR: Likelihood Ratio.

Depending on the sex distribution, the best cut-off value of TST among male participants (sensitivity: 77.2%; specificity: 75.2%; Youden Index: 0.524) and female participants (sensitivity: 70.7%; specificity: 77.9%; Youden Index: 0.486) was also ≥11 mm for both sex groups. The sensitivity became >80% from a TST cut-off value ≥10 mm to 5 mm and a TST cut-off value ≥8 mm to 5 mm, for male participants and female participants, respectively (see Table 4).

**Table 4.** Sensitivity, specificity, Youden index, positive and negative likelihood ratios values, corresponding to different possible tuberculin skin test thresholds (From 5-15 mm) by sex distribution (Male *versus* Female).

Diameter of TST induration ≥ (mm)	Sensitivity % [95% CI]		Specificity % [95% CI]		Youden index		Positive LR [95% CI]		Negative LR [95% CI]	
	Male	Female	Male	Female	Male	Female	Male	Female	Male	Female
5	90.5[84.8-94.6]	85.1 [79.0-89.9]	43.1[37.9-48.4]	47.5 [42.2-52.8]	0.336	0.325	1.6 [1.4-1.8]	1.6 [1.4-1.8]	0.22 [0.13-0.36]	0.31 [0.22-0.45]
6	89.2[83.3-93.6]	83.4 [77.2-88.5]	49.0[43.7-54.3]	52.8 [47.5-58.1]	0.382	0.362	1.7 [1,5-1.9]	1.8 [1.5-2.0]	0.22 [0.14-0.35]	0.31 [0.22-0.44]
7	87.3 [81.1-92.1]	82.9 [76.6-88.1]	53.8 [48.5-59.1]	57.5 [52.2-62.7]	0.411	0.404	1.9 [1.6-2.1]	2.0 [1.7-2.2]	0.23 [0.15-0.30]	0.29 [0.21-0.42]
8	86.7 [80.4-91.6]	81.8 [75.4-87.1]	57.5 [52.1-62.7]	60.6 [55.3-65.7]	0.441	0.423	2.0 [1.8-2.3]	2.1 [1.8-2.4]	0.23 [0.15-0.35]	0.30 [0.22-0.41]
9	83.5 [76.8-89.0]	79.0 [72.3-84.7]	63.1 [57.8-68.1]	65.9 [60.8-70.8]	0.466	0.449	2.3 [1.9-2.6]	2.3 [1.9-2.7]	0.26 [0.18-0.37]	0.32 [0.24-0.43]
10	82.9 [76.1-88.4]	76.2 [69.4-82.2]	67.6 [61.9-71.9]	68.4 [63.3-73.2]	0.499	0.446	2.5 [2.1-2.9]	2.4 [2.0-2.9]	0.25 [0.18-0.36]	0.35 [0.26-0.45]
**11**	**77.2** [69.9-83.5]	**70.7** [63.5-77.2]	**75.2** [70.4-79.6]	**77.9** [73.3-82.1]	**0.524**	**0.486**	**3.1** [2.5-3.8]	**3.2** [2.6-4.0]	**0.30** [0.23-0.40]	**0.37** [0.29-0.47]
12	72.2 [64.5-79.0]	66.8 [59.5-73.7]	78.0 [73.4-82.2]	80.2 [75.7-84.2]	0.501	0.470	3.3 [2.6-4.1]	3.4 [2.7-4.3]	0.36 [0.27-0.46]	0.41 [0.33-0.51]
13	62.0 [54.0-69.6]	60.8 [53.3-67.9]	82.3 [77.9-86.1]	83.8 [79.6-87.5]	0.442	0.445	3.5 [2.7-4.5]	3.8 [2.9-4.9]	0.46 [0.37-0.56]	0.47 [0.39-0.56]
14	51.9 [43.8-59.9]	57.5 [49.9-64.8]	86.8 [82.8-90.1]	86.3 [82.3-89.7]	0.386	0.437	3.9 [2.9-5.3]	4.2 [3.1-5.6]	0.55 [0.47-0.65]	0.49 [0.41-0.59]
15	46.8 [38.9-54.9]	54.1 [46.6-61.6]	88.2 [84.3-91.3]	88.5 [84.8-91.7]	0.350	0.426	3.9 [2.8-5.5]	4.7 [3.4-6.5]	0.60 [0.52-0.70]	0.52 [0.44-0.61]

TST: tuberculin skin test; CI: confidence interval; LR: Likelihood Ratio.

However, depending on the infection localisation, the best cut-off value for pulmonary TB (121 cases *versus* 714 controls) and lymph node TB (180 cases *versus* 714 controls) was also ≥11 mm, with an area under the curve (AUC) of 0.778 (95% CI: 0.733-0.822; p = 0.02) and 0.814 (95% CI: 0.777-0.851; p = 0.01), respectively (see Table 5, Figure 3B and C). The sensitivity became >80% from a TST cut-off value ≥8 mm to 5 mm and a TST cut-off value ≥10 mm to 5 mm, for pulmonary TB and lymph node TB, respectively (see Table 5).

**Table 5.** Sensitivity, specificity, Youden index, positive and negative likelihood ratios values, corresponding to different possible tuberculin skin test thresholds (From 5-15 mm) by infection type (pulmonary *versus* lymph node tuberculosis; n cases=301).

Diameter of TST induration ≥ (mm)	Sensitivity % [95% CI]		Specificity % [95% CI]		Youden index		Positive LR [95% CI]		Negative LR [95% CI]	
	Pulmonary	Lymph node	Pulmonary	Lymph node	Pulmonary	Lymph node	Pulmonary	Lymph node	Pulmonary	Lymph node
5	89.3 [82.4 – 93.6]	88.9 [83.4 – 92.6]	45.2 [41.6 – 48.9]	45.2 [41.4 – 48.7]	0.345	0.341	1.6 [1.5 – 1.8]	1.6 [1.5 – 1.8]	0.22 [0.13 – 0.38]	0.25 [0.16 – 0.37]
6	86.0 [78.6 – 91.0]	88.9 [83.4 – 92.6]	50.8 [47.1 – 54.4]	50.8 [47.0 – 54.3]	0.368	0.397	1.7 [1.6 – 1.9]	1.8 [1.6 – 2.0]	0.28 [0.18 – 0.43]	0.22 [0.14 – 0.33]
7	83.5 [75.8 – 89.0]	88.3 [82.8 – 92.2]	55.6 [51.9 – 59.2]	55.6 [51.9 – 59.2]	0.391	0.439	1.9 [1.7 – 2.1]	2.0 [1.8 – 2.2]	0.30 [0.20 – 0.45]	0.21 [0.14 – 0.32]
8	83.5 [75.8 – 89.0]	87.2 [81.5 – 91.3]	58.9 [55.3 – 62.5]	58.9 [55.3 – 62.5]	0.424	0.461	2.0 [1.8 – 2.3]	2.1 [1.9 – 2.4]	0.28 [0.19 – 0.42]	0.22 [0.15 – 0.32]
9	78.5 [70.3 – 84.8]	85.0 [79.0 – 89.4]	64.4 [60.8 – 67.8]	64.4 [60.8 – 67.8]	0.429	0.494	2.2 [1.9 – 2.5]	2.4 [2.1 – 2.7]	0.33 [0.24 – 0.47]	0.23 [0.16 – 0.33]
10	76.9 [68.5 – 83.4]	83.9 [77.8 – 88.5]	67.6 [64.1 – 70.9]	67.6 [64.1 – 70.9]	0.445	0.515	2.4 [2.0 – 2.8]	2.6 [2.3 – 2.9]	0.34 [0.25 – 0.48]	0.24 [0.17 – 0.33]
**11**	**70.2** [61.5 – 77.6]	**77.8** [71.1 – 83.2]	**76.6** [73.3 – 79.5]	**76.6** [73.3 – 79.5]	**0.468**	**0.544**	**3.0** [2.5 – 3.6]	**3.3** [2.8 – 3.9]	**0.39** [0.29 – 0.51]	**0.29** [0.22 – 0.38]
12	65.3 [56.4 – 73.1]	73.3 [66.4 – 79.2]	79.1 [76.0 – 81.9]	79.1 [76.0 – 81.9]	0.444	0.524	3.1 [2.6 – 3.8]	3.5 [3.0 – 4.2]	0.44 [0.34 – 0.56]	0.34 [0.26 – 0.43]
13	57.9 [48.9 – 66.3]	64.4 [57.2 – 71.1]	83.1 [80.1 – 85.6]	83.1 [80.1 – 85.6]	0.410	0.475	3.4 [2.7 – 4.3]	3.8 [3.1 – 4.6]	0.51 [0.41-0.63]	0.43 [0.35 – 0.52]
14	51.2 [42.4 – 60.0]	59.4 [52.1 – 66.3]	86.6 [83.9 – 88.9]	86.6 [83.9 – 88.9]	0.378	0.460	3.8 [2.9 – 4.9]	4.4 [3.5 – 5.5]	0.56 [0.47 – 0.68]	0.47 [0.39 – 0.56]
15	47.9 [39.2 – 56.8]	54.4 [47.1 – 61.5]	88.4 [85.8 – 90.5]	88.4 [85.8 – 90.5]	0.363	0.428	4.1 [3.1 – 5.4]	4.7 [3.7 – 6.0]	0.59 [0.50 – 0.70]	0.51 [0.44 – 0.60]

TST: tuberculin skin test; CI: confidence interval; LR: Likelihood Ratio.

Depending on age groups, the best cut-off value of TST among participants aged <35 years old (n = 148 cases; sensitivity: 79.1%; specificity: 79.8%; Youden Index: 0.589; AUC: 0.822 (95% CI: 0.782 – 0.862; p = 0.02)) and participants aged ≥35 years (n = 188 cases; sensitivity: 70.2%; specificity: 71.3%; Youden Index: 0.415; AUC: 0.750 (95% CI: 0.703 – 0.798; p = 0.02)) was also ≥11 mm for both groups.

## Discussion

TB remains a serious public health threat. In this study, we evaluated the performance of TST in a diagnostic situation and we measured its different accuracy metric indicators for different possible cut-off points, among a large size population, with 1050 participants. The best discriminating cut-off value was chosen based on the best couple sensitivity and specificity with the highest Youden Index value.

The TST is widely used for the detection of a latent TB form and for screening the contacts of a TB patient. This test has the advantage of being easy, rapid and safe to conduct, and low-cost. ^20^

In view of our results, it may be concluded that the TST can be used for active TB diagnosis with a moderate global performance (AUC = 0.789), ^19^^,^^21^ and a good couple of sensitivity and specificity at the cut-off point of 11 mm.

The results of the positive (3.1) and negative (0.34) LR of the selected cut-off value also indicates a moderate performance and utility of the TST. ^22^

There is no international agreement about the best cut-off value of the TST which clearly distinguishes between a positive and negative test result. Different suggestions were proposed varying between 5mm and 10mm diameter of induration. But it depends on many epidemiological and immunological patient risk factors. ^23^

Considering our results, the sensitivity improved (>80%) from a TST cut-off value ≥9 mm to 5 mm. So, the induration diameter threshold of TST can be reduced from 11mm (the best cut-off value) to 9mm to enhance the sensitivity of TST and consequently, reduce the frequency of false negatives and limit the miss-diagnosis of a tuberculosis infection case, regardless of its pulmonary or extra-pulmonary form.

Moreover, the challenging difficulties encountered in diagnosing extrapulmonary TB, which is requiring invasive diagnostic procedures, poses a diagnostic problem for clinicians. ^24^

In Tunisia, diagnostic confirmation of lymph node TB requires needle aspiration or surgical excision with cytological or pathological and bacteriological study (direct examination, Xpert MTB/RIF test and culture). Generally, the lymph node TB lesions are paucibacillary with a rarely positive direct examination (about 10% of positive rate only). ^11^

Since lymph node tuberculosis is usually more difficult to clinically diagnose than pulmonary tuberculosis, it is thus possible, in case of lymph node tuberculosis suspicion, to use the 10 mm threshold to guarantee a good sensitivity (>80%) of the test.

However, the TST has some limitations. the skin induration measurement is operator-dependent and remains subjective; its interpretation depends on many other factors, like the immunological status, co-morbid conditions, and prior Calmette Guerin (BCG) vaccination. ^20^^,^^25^

Indeed, the TST is not a specific test that distinguish between TB infection and post-vaccination allergy. The cross-reaction of TST with antigens of other non-tuberculous mycobacteria and with the antigen used for the Calmette Guerin (BCG) vaccine generates false positives, inducing an apparent false increase in sensitivity and a decrease in specificity. ^3^^,^^26^

Certainly, progress has been made in developing other rapid tests for TB screening like Xpert MTB/RIF, Xpert MTB/RIF Ultra ^2^ and Interferon-gamma release assay (IGRA) tests. However, they are still often not available and expensive, especially in low- and middle-income countries, which have the highest TB burden. ^3^^,^^27^ That is why these novel tests are not an accessible and available option for TB diagnosis in these unfavorable areas. ^27^

The strengths of our study include the large sample size of included participants (1050), the easy application of the TST and the clear and detailed description of the diagnostic test evaluation methodology, for further repeatability. As any epidemiological study, there were some limitations, essentially including the sample of controls which may not be representative of all non-TB participants the real life, and the non-blinded administration of the test, which may influence the operator interpretation of the test result.

Therefore, for more suitable evaluation of the TST performance for diagnosing active TB, further prospective research studies are needed. We propose to follow-up for a minimum two years, and a sample of closely exposed contacts of TB patients, for prospective detection of incident newly infected cases. At the end of the follow-up period, the contact subjects will be divided into newly diagnosed active cases and controls, which represent a nested case-control study. This method will minimize the selection bias for a good representativeness of included cases and controls. All included participants will receive the TST and the IGRA test, at the same time. It will be therefore possible to compare the performance of the two tests by comparing their corresponding ROC curves.

A progressive diagnostic approach based on a flowchart decision strategy using consecutive tests can also be a good alternative. The TST can be used as the first line test because it is the easiest and cheapest test to apply at a large scale (with good sensitivity); then, considering TST results, the IGRA test can be used in second line for more specific results. ^28^^–^^30^

Economic evaluation of the cost-effectiveness of this diagnostic approach must be conducted to support the usefulness and the rational application of this strategy for public health purposes.

Finally, the ROC curve certainly has an important role in determining the best TST cut-off for the TB diagnosis or screening. However, it is important to consider that the interpretation of the TST characteristics is based on probabilities. Also, its predictive values cannot be properly assessed without considering all other risk factors. So, there is a need to develop a predictive clinical score for TB based on a range of epidemiological (TB prevalence, demographic characteristics, notion of contagion), clinical (individual risk factors, TB symptoms) and paraclinical examinations (chest X-ray, among others) factors. This approach can optimize and guide the use of the various diagnostic methods for TB. Unnecessary invasive examinations and procedures will be, therefore, avoided while reducing TB diagnosis cost.

## Conclusions

The TST is simple to perform and a low-cost test. It can be used for active TB detection with a moderate global performance and accepted sensitivity and specificity at the cut-off point of 11mm. However, it has some disadvantages, especially its low specificity regarding high rates of false positives in areas with mass BCG vaccination. The association of the TST with another test such as the IGRA test would be a good alternative for early and accurate diagnosis of TB.

## Data Availability

Harvard Dataverse: Tuberculin Skin Test, https://doi.org/10.7910/DVN/630CJG This project contains the following underlying data:
•
TST-final.tab (anonymised underlying data collected from 1050 included patient) Data are available under the terms of the Creative Commons Zero “No rights reserved” data waiver (CC0 1.0 Public domain dedication).
