# Supplementary material for: How useful is the tuberculin skin test for tuberculosis detection: Assessing diagnostic accuracy metrics through a large Tunisian case-control study
Source: F1000Res. 2024 Jul 1;12:1297. Originally published 2023 Oct 10. [Version 2] doi: 10.12688/f1000research.138211.2 (PMC11274051; doi:10.12688/f1000research.138211.2)
Supplement: Peer review history

# Reviewer report: Reviewer response for version 2

**DOI:** 10.5256/f1000research.168401.r297793

**Published:** 2024-07-24

**Authors:** Oliver Stirrup

**Affiliations:** 1 University College London, London, UK

Some requested changes have been made, although the authors have not incorporated all of their responses to my previous comments into the main text. I felt that the responses to my queries would be useful clarifications to the article, but will not insist on further amendments. I also still feel that information is lacking regarding the variable coding for the raw data provided.

Is the work clearly and accurately presented and does it cite the current literature?

Partly

If applicable, is the statistical analysis and its interpretation appropriate?

Yes

Are all the source data underlying the results available to ensure full reproducibility?

Partly

Is the study design appropriate and is the work technically sound?

Partly

Are the conclusions drawn adequately supported by the results?

Partly

Are sufficient details of methods and analysis provided to allow replication by others?

Partly

Reviewer Expertise:

Medical Statistician, with limited experience of TB research

I confirm that I have read this submission and believe that I have an appropriate level of expertise to confirm that it is of an acceptable scientific standard.

# Reviewer report: Reviewer response for version 2

**DOI:** 10.5256/f1000research.168401.r297794

**Published:** 2024-07-15

**Authors:** Paula Rodríguez-Molino

**Affiliations:** 1 Hospital La Paz, La Paz Research Institute (IdiPAZ), Universidad Autónoma de Madrid (UAM), Madrid, Spain; 2 Centro de Investigacion Biomedica en Red Enfermedades Infecciosas (Ringgold ID: 637284), Madrid, Community of Madrid, Spain

I agree with the changes made and approve the manuscript is ready for indexing.

Is the work clearly and accurately presented and does it cite the current literature?

Yes

If applicable, is the statistical analysis and its interpretation appropriate?

Yes

Are all the source data underlying the results available to ensure full reproducibility?

Yes

Is the study design appropriate and is the work technically sound?

Yes

Are the conclusions drawn adequately supported by the results?

Yes

Are sufficient details of methods and analysis provided to allow replication by others?

Yes

Reviewer Expertise:

NA

I confirm that I have read this submission and believe that I have an appropriate level of expertise to confirm that it is of an acceptable scientific standard.

# Reviewer report: Reviewer response for version 1

**DOI:** 10.5256/f1000research.151395.r266923

**Published:** 2024-05-10

**Authors:** Paula Rodríguez-Molino

**Affiliations:** 1 Hospital La Paz, La Paz Research Institute (IdiPAZ), Universidad Autónoma de Madrid (UAM), Madrid, Spain; 2 Centro de Investigacion Biomedica en Red Enfermedades Infecciosas (Ringgold ID: 637284), Madrid, Community of Madrid, Spain

The authors propose a Case-control study to analyze the performance of TST for active TB. I extend my sincere congratulations to them for conducting this study with such a large cohort of patients, which is of great utility. The future study they propose in a nested case-control cohort is interesting, and I encourage them to also include pediatric patients.

 In general, I recommend reviewing English grammar.

**- Title:** Consider tuberculosis disease instead of active TB, and TB infection instead of latent TB, according to the latest terminology. Also, consider shortening the title length.

**- Introduction**

 I suggest including a reference in 6 ^th^ paragraph, referring to Pulmonary and extrapulmonary TB forms in Tunisia.

**- Methods:**

 Methods section is well explained, easily allowing replication.

 I just wanted to point out a grammar mistake in the “Sensitivity and specificity”, 10 ^th^ paragraph: “the TST a test is more discriminating”.

 * ***Ethical considerations***: Another grammar mistake in 1 ^st^ pg, it should be “have given”

 * ***Study populations***

- The part that generates the most doubts for me is the definition of cases and controls.
- I understand that not all cases were microbiologically confirmed. If not, please specify how many had microbiological confirmation.
- In the case of patients with adenitis, did you rule out the possibility of non-tuberculous mycobacteria that could have cross-reacted with the TST?
- Regarding the controls, how did you ensure they were free of TB? Did they have chest X-rays, or was it based solely on the absence of symptoms compatible with TB? Since in the controls the TST was performed as part of routine clinical practice and not as part of the study, Was TB infection ruled out in those cases that had a TST >5-10mm? On the other hand, if TST was done as part of contact tracing, Was it repeated after 8-12 weeks? How did you differentiate if the controls were free of TB or latent TB infection?

** Results**

*TABLE 1: I recommend that you specify the other sites of extrapulmonary TB

 *Did you analyze the time between the contact risk or the beginning of the symptoms and TST performance?

 -*Did you analyze the reliability of the test according to the severity of the disease?

Is the work clearly and accurately presented and does it cite the current literature?

Yes

If applicable, is the statistical analysis and its interpretation appropriate?

Yes

Are all the source data underlying the results available to ensure full reproducibility?

Yes

Is the study design appropriate and is the work technically sound?

Yes

Are the conclusions drawn adequately supported by the results?

Yes

Are sufficient details of methods and analysis provided to allow replication by others?

Yes

Reviewer Expertise:

Pediatric Infectious and Tropical Diseases

I confirm that I have read this submission and believe that I have an appropriate level of expertise to confirm that it is of an acceptable scientific standard, however I have significant reservations, as outlined above.

# Response

**Published:** 2024-06-25

**Authors:** Mariem Nouira

**Affiliations:** Faculty Of Medicine Of Tunis-Tunisia, Tunisia

Dear Dr Paula Rodríguez-Molino,

 Thank you very much for your review. We really appreciate your response and time. We have revised our manuscript as you suggested. Here are the responses to your comments:

**Reviewer Comment:** 

 - Title: Consider tuberculosis disease instead of active TB, and TB infection instead of latent TB, according to the latest terminology. Also, consider shortening the title length. 

***Author Response:*** It was done. Thank you

 “how useful is the tuberculin skin test for tuberculosis detection: Assessing diagnostic accuracy metrics through a large Tunisian case-control study”

**Reviewer Comment:** 

 - Introduction

 I suggest including a reference in 6th paragraph, referring to Pulmonary and extrapulmonary TB forms in Tunisia.

***Author Response:*** The ref 11 corresponds to the entire previous paragraph (6th paragraph)

**Reviewer Comment:** 

 - Methods:

 I just wanted to point out a grammar mistake in the “Sensitivity and specificity”, 10th paragraph: “the TST a test is more discriminating”. Response: it was corrected. Thank you

 *Ethical considerations: Another grammar mistake in 1st pg, it should be “have given”

***Author Response:*** it was corrected. Thank you.

**Reviewer Comment:** 

 •    I understand that not all cases were microbiologically confirmed. If not, please specify how many had microbiological confirmation.

***Author Response:*** Unfortunately, the information regarding microbiological confirmation was not collected. This was a selection criteria that was applicated to include cases and was not noted in the data collection 

**Reviewer Comment:** 

 •    In the case of patients with adenitis, did you rule out the possibility of non-tuberculous mycobacteria that could have cross-reacted with the TST?

***Author Response:*** ​​​​​​​ Yes. The TB diagnosis was not based on a positive result of TST. The TB diagnosis was confirmed based on a range of anamnestic, clinical, chest X-ray and bacteriological arguments. 

**Reviewer Comment:** 

 •    Regarding the controls, how did you ensure they were free of TB? Did they have chest X-rays, or was it based solely on the absence of symptoms compatible with TB?

***Author Response:*** ​​​​​​​ The TB diagnosis was not only based on the absence of TB symptoms. The TB diagnosis was confirmed based on a range of anamnestic, clinical, chest X-ray and bacteriological arguments. 

**Reviewer Comment:** 

 •    Since in the controls the TST was performed as part of routine clinical practice and not as part of the study, Was TB infection ruled out in those cases that had a TST >5-10mm?

***Author Response:*** ​​​​​​​ Yes, the diagnosis was not based on the result of the TST. Many other factors were taken into account to confirm the TB infection 

**Reviewer Comment:** 

 •    On the other hand, if TST was done as part of contact tracing, Was it repeated after 8-12 weeks? How did you differentiate if the controls were free of TB or latent TB infection?

***Author Response:*** ​​​​​​​ No we didn’t collect information regarding the repeat of TST. We only collected information regarding the first TST done.

**Reviewer Comment:** 

  Results

 *TABLE 1: I recommend that you specify the other sites of extrapulmonary TB.

***Author Response:*** ​​​​​​​  It was done. Thank you

**Reviewer Comment:** 

 *Did you analyze the time between the contact risk or the beginning of the symptoms and TST performance?

***Author Response:*** ​​​​​​​No, unfortunately we did not analyse this information 

**Reviewer Comment:** 

 -*Did you analyze the reliability of the test according to the severity of the disease?

***Author Response:*** No, unfortunately we did not analyse this information

# Reviewer report: Reviewer response for version 1

**DOI:** 10.5256/f1000research.151395.r257311

**Published:** 2024-04-22

**Authors:** Oliver Stirrup

**Affiliations:** 1 University College London, London, UK

Nouira *et al.* describe a case-control study evaluating the diagnostic performance of the tuberculin skin test (TST) for active TB. I am a statistician with only limited experience of TB research. As such, I have commented on the statistical methods used and the study design, but not on the scientific novelty and interest of this work.

 The statistical methods that have been applied are appropriate for the data, and have been described and interpreted correctly. I only have a minor comment regarding the statistical elements:

 -Methods: “Positive and negative predictive values (PPV and NPV) (or post-test probabilities) of TST were also determined for different possible TST thresholds by two different methods, using the properties of Bayes’ theorem and using Fagan’s nomogram graph”. Fagan’s nomogram is not really a ‘different method’, but rather a graphical application of Baye’s theorem.

 My main concern in reading this manuscript is the description of the recruitment and selection of the cases and controls, which for me leaves a number of questions unanswered:

 -Did you approach/include all people who underwent the TST at the participating centers during the time period stated?

 -No details are given regarding the number of cases and controls excluded due to the exclusion criteria.

 -Did anyone decline involvement?

 -Was there any particular reason to evaluate the specific time period chosen?

 -Why did the cases undergo a TST at the time of their anti-TB treatment?

 -Why did the controls undergo a TST if they had no signs of TB?

 -Was the data collection questionnaire filled in by participants? Or was it filled in retrospectively by clinics?

 -Were participants recruited prospectively at each clinic? This is implied but not explicitly stated.

 Further minor points:

 -The English writing throughout can be understood, but would benefit from further review and editing.

 -I don’t think that there is much value in Figure 2, unless the TST values are divided by both sex and case-control status.

 -Some of the references cited do not have full details listed (although you can click through the hyperlinks for the intended target documents), e.g. 1 and 2.

 -Anonymized underlying data have been provided, but the data coding is not clear for all variables.

 -The data described are 10 years old. Are there likely to have been further changes to TB epidemiology in this time?

Is the work clearly and accurately presented and does it cite the current literature?

Partly

If applicable, is the statistical analysis and its interpretation appropriate?

Yes

Are all the source data underlying the results available to ensure full reproducibility?

Partly

Is the study design appropriate and is the work technically sound?

Partly

Are the conclusions drawn adequately supported by the results?

Partly

Are sufficient details of methods and analysis provided to allow replication by others?

Partly

Reviewer Expertise:

Medical Statistician, with limited experience of TB research

I confirm that I have read this submission and believe that I have an appropriate level of expertise to confirm that it is of an acceptable scientific standard, however I have significant reservations, as outlined above.

# Response

**Published:** 2024-06-25

**Authors:** Mariem Nouira

**Affiliations:** Faculty Of Medicine Of Tunis-Tunisia, Tunisia

Dear Dr Oliver Stirrup,

 Thank you very much for your review. We really appreciate your response and time. We have revised our manuscript as you suggested. Here are the responses to your comments:

**Reviewer Comments:**

 •    Fagan’s nomogram is not really a ‘different method’, but rather a graphical application of Baye’s theorem.

***Author Response:*** It was corrected Thank you. 

**Reviewer Comments:**

 •    Did you approach/include all people who underwent the TST at the participating centers during the time period stated?

***Author Response:*** Yes, we included all people who underwent the TST at the participating centers during the time period stated.

**Reviewer Comments:**

 •    No details are given regarding the number of cases and controls excluded due to the exclusion criteria.  

***Author Response:*** Unfortunately, we do not know their exact number because they were not compatibilized. 

**Reviewer Comments:**

 •    Did anyone decline involvement?

***Author Response:*** No one declined involvement 

**Reviewer Comments:**

 •    Was there any particular reason to evaluate the specific time period chosen?

***Author Response:*** No there was not any particular reason for the study period. 

**Reviewer Comments:**

 •    Why did the cases undergo a TST at the time of their anti-TB treatment?

***Author Response:*** No they did not undergo a TST at the time of their anti-TB treatment. They were recruited at the time of their anti-TB treatment and the TST was done before recruitment. 

**Reviewer Comments:**

 •    Why did the controls undergo a TST if they had no signs of TB?

***Author Response: *** Controls undergo a TST in the context of contact tracing or to eliminate a TB diagnosis based on a range of anamnestic, radiological and bacteriological arguments. 

**Reviewer Comments:**

 •    Was the data collection questionnaire filled in by participants? Or was it filled in retrospectively by clinics?

***Author Response:*** It was filled in retrospectively by investigators 

**Reviewer Comments:**

 •    Were participants recruited prospectively at each clinic? This is implied but not explicitly stated.  

***Author Response: *** Yes they were recruited prospectively but the data collection regarding the TST was collected retrospectively

**Reviewer Comments:**

 •    I don’t think that there is much value in Figure 2, unless the TST values are divided by both sex and case-control status.

***Author Response:*** Figure 2 was eliminated. Thank you

**Reviewer Comments:**

 •    Some of the references cited do not have full details listed (although you can click through the hyperlinks for the intended target documents), e.g. 1 and 2.

***Author Response:*** It was corrected. Thank you

**Reviewer Comments:**

 •    Anonymized underlying data have been provided, but the data coding is not clear for all variables.

***Author Response:*** Thank you. It was done. The underlying data have been updated . 

**Reviewer Comments:**

 •    The data described are 10 years old. Are there likely to have been further changes to TB epidemiology in this time?

***Author Response:*** Yes it is possible that changes have been occured on TB epidemiology. It may be a limit of our study.
